# Effect of Aberrant Long Noncoding RNA on the Prognosis of Clear Cell Renal Cell Carcinoma

**DOI:** 10.1155/2021/6533049

**Published:** 2021-09-03

**Authors:** Han Wu, Haixiao Wu, Peng Sun, Desheng Zhu, Min Ma, Wentao Fan

**Affiliations:** Department of Urology Surgery, Jinhua Municipal Central Hospital, Jinhua 321001, China

## Abstract

Clear cell renal cell carcinoma (ccRCC) is a kind of lethal cancer. Although there are mature treatment methods, there is still a lack of rigorous and scientific means for cancer diagnosis. Long noncoding RNAs (lncRNAs) are a kind of noncoding RNA (ncRNA). Recent studies find that alteration of lncRNA expression is related to the occurrence of many cancers. In order to find lncRNAs which can effectively predict the prognosis of ccRCC, RNA-seq count data and clinical information were downloaded from TCGA-KIRC, and gene expression profiles from 530 patients were included. Then, *K*-means was used for clustering, and the number of clusters was determined to be 5. The R-package “edgeR” was used to perform differential expression analysis. Subsequently, a risk model composed of 10 lncRNA biomarkers significantly related to prognosis was identified via Cox and LASSO regression analyses. Then, patients were divided into two groups according to the model-based risk score, and then, GSEA pathway enrichment was performed. The results showed that metabolism- and mTOR-related pathways were activated while immune-related pathways were inhibited in the high-risk patients. Combined with previous studies, it is believed that these 10 lncRNAs are potential targets for the treatment of ccRCC. In addition, Cox regression analysis was used to verify the independence of the risk model, and as results revealed, the risk model can be used to independently predict the prognosis of patients. In conclusion, our study found 10 lncRNAs related to the prognosis of ccRCC and provided new ideas for clinical diagnosis and drug development.

## 1. Introduction

Renal cell carcinoma (RCC) is the most common cancer in the kidney of adults, and the incidence has been increasing in the past decades [1]. The tumor tissue of ccRCC patients often comes with bleeding, necrosis, cystic change, and calcification. With the progression of cancer, it will form hemangioma thrombus and even metastasize to lymph nodes and other organs. Since ccRCC has strong resistance to chemotherapy and radiotherapy, the main treatment for ccRCC is surgery, and partial nephrectomy is the most effective and commonly used treatment so far [2, 3]. Although most ccRCC patients can be cured with surgical treatment, there are still 30% of ccRCC patients developing tumor metastasis after surgery [4, 5]. Many studies have been involved in the molecular mechanisms of ccRCC. For example, Yin and other investigators found that NR1B2 can inhibit the development of ccRCC by regulating LATS 1/2-YAP signaling pathway [6]. Hakimi and other experts found that mutations in two epigenetic regulators on chromosome 3p21, BAP1 and SETD2, can affect the progression of ccRCC [7]. However, there is still a lack of biomarkers to guide clinical diagnosis and treatment of ccRCC. Therefore, it is very important to further research the molecular mechanisms of ccRCC to guide the development of therapeutic drugs and clinical diagnosis.

lncRNA is a kind of RNA that cannot be translated into proteins. Similar to protein-coding transcripts, lncRNA transcripts are processed by spliceosome mechanisms [8–11]. While being compared with protein-coding genes, lncRNA coding genes are composed of fewer exons and are less selective and less abundant in the evolution [12]. When being transcribed from the enhancer region or adjacent sites, lncRNAs can be used as scaffolds or guides for regulating protein-protein or protein-DNA interactions, as a bait for binding to proteins or miRNAs, and as an enhancer influencing gene transcription [13–18]. Recent studies suggested that the expression of lncRNA affects the development of tumors, and it plays a role as a tumor suppressor or promoter with its alteration in transcriptional level in tumor tissue. For example, Zhai and other experts found that there is a feedback loop between lncRNA-URRCC and EGFL7/p-AKT/FOXO3 signals, which promotes the proliferation and metastasis of ccRCC [19]. In addition, some studies also found that HOTAIR can promote the invasion of cervical cancer by targeting Notch pathway and HULC can promote the metastasis of hepatocellular carcinoma through miR-200a-3p/ZEB1 signaling pathway [20, 21]. In addition to direct impacts, lncRNA is also discovered to play a key regulatory role in many biological processes of cancers. For example, lncRNA participates in the regulation, transcription, and posttranscriptional processes of chromatin state [17, 22–24]. In recent years, with the development of high-throughput detection techniques and bioinformatics analysis, the construction of cancer prognosis model based on lncRNA has become a mainstream. A number of studies have mined prognostic biomarkers for different cancers based on the expression data of lncRNAs using various bioinformatics analyses. For instance, Sun et al. established a prognostic model based on autophagy-associated lncRNA for bladder urothelial cancer [25]. Likewise, Lian et al. [26] explored lncRNAs associated with prognosis of bladder cancer patients from public databases. All in all, bioinformatics analysis is more effective to find prognostic biomarkers of specific cancer from complicated lncRNA expression data in comparison with traditional methods.

In this study, lncRNA expression matrix and matched clinical information of ccRCC patients were downloaded from TCGA and analyzed with bioinformatics methods. Firstly, *K*-means clustering was used to classify the patients, and then univariate Cox, LASSO, and multivariate Cox regression models were used to further screen lncRNAs related to the prognosis of ccRCC. A risk model based on the identified lncRNAs was then established, and the lncRNAs were noted to be related to metabolism, immunity and epithelial-mesenchymal transition (EMT). In conclusion, we found 10 lncRNA biomarkers related to the prognosis of ccRCC and further understood the molecular mechanisms underlying the development of ccRCC, which provides new ideas and experimental basis for the diagnosis and treatment of ccRCC patients.

## 2. Materials and Methods

### 2.1. Data Downloading and Processing

Transcriptome expression matrix data and matched clinical data of ccRCC patients were downloaded from TCGA (https://portal.gdc.cancer.gov/) on December 20, 2019. Then, sequencing data of samples from 530 ccRCC patients with complete clinical information (Supplementary Table 1) were obtained, and the samples were randomly divided into the training set and the test set at 7 : 3. lncRNAs were annotated with human genome annotation document downloaded from the GENCODE database (https://www.gencodegenes.org/) and used for subsequent analyses.

### 2.2. lncRNA *K*-Means Clustering and Patient Grouping

Firstly, lncRNA expression data in the training set were standardized using scale function. The training set data were used to determine cluster number with the elbow method, and then, the patients were classified by the *K*-means method according to the lncRNA expression profile. Finally, the patients were grouped according to the clustering results [27, 28]. In *K*-means clustering, the sum of square-error between the centroid and each data point in a cluster was calculated to defined distortion degree, which decreased with the increase of cluster number. For a dataset with certain discrimination, the distortion degree was greatly improved when it reached a certain critical point, and then, it decreased slowly. This critical point can be considered the point with good clustering performance. The best clustering number can be determined with an experimental line graph based on the distortion degree and cluster number, and then *K*-means clustering can be carried out according to the determined cluster number.

### 2.3. Differential Expression Analysis

Patients were divided into groups according to the clustering results. The differences in gene expression between the samples in one cluster and the samples in the other clusters in the training set were analyzed (log  | FC | >1, FDR < 0.05) with the R-package edgeR [29, 30]. Differential lncRNAs in each cluster were finally obtained. The results were visualized with heat map.

### 2.4. Identification of ccRCC Prognosis-Related Genes

Following differential expression, survival package [31] was used to perform univariate Cox regression analysis to explore the differentially expressed lncRNAs of all clusters, and the lncRNAs significantly related to clinical risk of ccRCC were screened. Then, LASSO regression analysis was conducted to screen redundant prognosis-related genes using glmnet package [32]. Finally, lncRNAs which had significant impact on the prognosis of ccRCC patients were screened out.

### 2.5. Construction and Verification of Risk Model

Prognosis-associated genes were obtained after LASSO regression analysis, and then, survival package was used for multivariate Cox regression analysis. A risk model was then established, and samples in the training set and the test set were evaluated with the expression level of signature genes and corresponding risk coefficient. According to the median risk score in all samples, the samples were divided into the high-risk group and low-risk group. The difference in survival between the two groups was shown with Kaplan-Meier curves. Then, in order to evaluate the accuracy and predictive value of the risk model, time-dependent ROC curves for 1-year, 3-year, and 5-year survivals were drawn to obtain the area under the curve (AUC) value. The model was validated in both the training set and the test set.

### 2.6. Model Independence Verification

In order to verify the independence of the constructed lncRNA-based risk model in the risk prediction of ccRCC, traditional clinical characteristics of ccRCC and risk values calculated by the model were subjected to Cox regression analysis using survival package.

### 2.7. Establishment of Prognostic Nomogram

R-package “rms” [33] was used to draw a nomogram based on the above factors, and fitting curves presenting the predicted results and the actual survival situation were drawn. The nomogram was used to help clinicians to evaluate the survival time of patients.

### 2.8. Functional Analysis

In order to study the potential mechanisms of the screened genes affecting the prognosis of ccRCC patients, GSEA enrichment analysis was carried out in the high- and low-risk groups, and the enrichment results were analyzed with *p* < 0.05 as the threshold [34, 35]. GSEA enrichment analysis was used to evaluate whether different metabolic pathways are enriched in different samples, and finally, the differences in gene sets of interest among different samples can be achieved.

### 2.9. Data Analysis

Unless specified, the threshold of significance in this study was FDR < 0.05, and all data in the research were displayed in the mode of mean ± SD (standard deviation). Experimental results were calculated and visualized with GraphPad Prism 6 and R (3.5.0) software.

## 3. Results

### 3.1. *K*-Means Clustering and Identification of Differentially Expressed lncRNA

**Figure 1:**
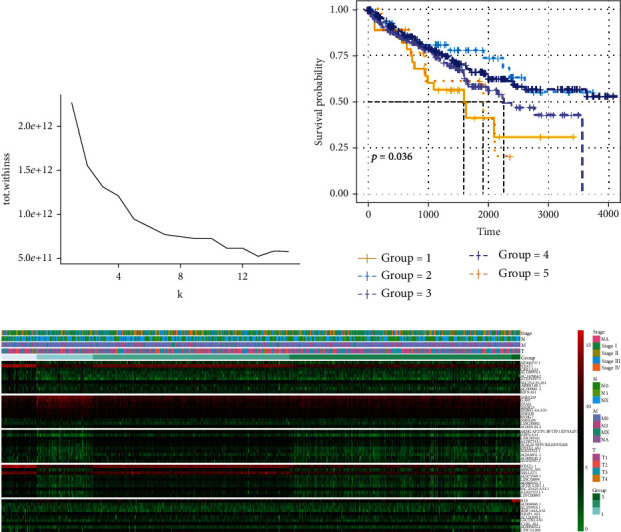
*K*-means clustering and differential gene expression analysis. (a) *K*-means clustering of the expression matrix of lncRNA; (b) overall survival (OS) for each cluster (*p* = 0.036); (c) heat map of differentially expressed lncRNAs in each cluster.

Firstly, *K*-means clustering was performed on the lncRNA expression profile of ccRCC patients to determine the optimal cluster number. The degree of distortion with the cluster number set from 1 to 15 was calculated. It can be seen from the figure that the distortion degree before the cluster number being 5 was greatly improved and then decreased after 5. Therefore, cluster number was determined to be 5 (Figure 1(a)). After clustering, survival analysis was performed on corresponding samples in the 5 clusters. The results of the *K*-means clustering were significantly related to the survival of patients, indicating that the *K*-means clustering can judge disease severity based on lncRNA expression profile and classify the patients (Figure 1(b)). In order to further screen out differentially expressed lncRNAs, a differential expression analysis was performed on the samples in one cluster and the samples in the other clusters. Finally, there were 95 differentially expressed lncRNAs in cluster 1, 62 in cluster 2, 22 in cluster 3, 22 in cluster 4, and 84 in cluster 5. According to the significance of the differential expression, ten genes with the most significant difference were selected from each cluster to draw a heat map (Figure 1(c)). The above results showed that the *K*-means clustering effect is good, and there are significant differences in the expression of lncRNAs among various clusters.

### 3.2. Screening of Prognosis-Related lncRNAs and Construction of Risk Model

**Figure 2:**
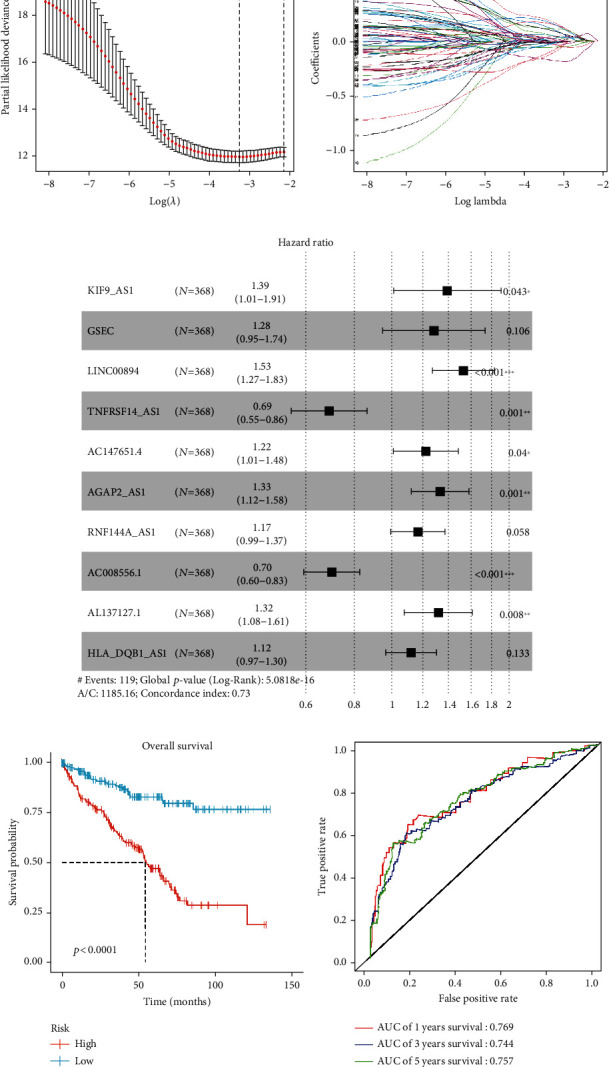
Screening of prognosis-related lncRNAs and construction of risk model. (a) 10-time cross-validation for tuning parameter selection in the LASSO model; (b) LASSO coefficient profiles of 75 prognosis-related lncRNAs; (c) multivariate Cox regression analysis of the association between 10 lncRNAs and OS of patients in the training set; (d) OS curves of ccRCC patients in the training cohort according to the 10-lncRNA-based risk model (*p* < 0.0001); (e) time-dependent ROC curves for 1-year, 3-year, and 5-year survivals of patients in the training set.

**Figure 3:**
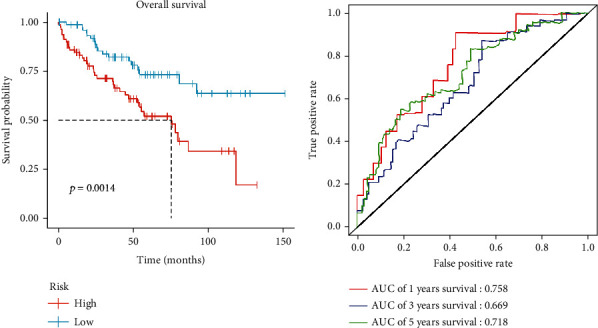
Validation of the 10-lncRNA-based risk model. (a) OS curves of ccRCC patients in the test cohort (*p* = 0.0014); (b) time-dependent ROC curves for 1-year, 3-year, and 5-year survivals of patients in the test set.

Univariate Cox regression analysis was used to analyze the differentially expressed lncRNAs screened, and 75 prognosis-related genes were obtained. Then, LASSO regression analysis was used to further screen these lncRNAs. Thereafter, 18 relatively independent prognosis-related lncRNAs were selected for subsequent model construction (Figures 2(a) and 2(b)). Finally, the 18 lncRNAs obtained were further analyzed with multivariate Cox step regression, and 10 lncRNAs (KIF9_AS1, GSEC, LIN00894, TNFRSF14_AS1, AC147651.4, AGAP2_AS1, RNF144A_AS1, AC008556.1, AL137127.1, and HLA_DQB1_AS1) which were significantly associated with prognosis were eventually identified, and a 10-lncRNA-based risk model (Figure 2(c)) was constructed. For validation, the samples in the training set were divided into the high-risk group and low-risk group according to the median risk score. The Kaplan-Meier method was used to compare the survival time in high/low-risk groups. log-rank test was used for significance test, and OS curves were drawn. The results showed that the OS rate in the high-risk group was significantly lower than that in the low-risk group (Figure 2(d)). ROC curves were used to evaluate the 1-year, 3-year, and 5-year survival times of the patients. The results showed that AUC values of the three groups were all greater than 0.7, demonstrating that the risk model was accurate in predicting the prognosis of patients with ccRCC (Figure 2(e)). The test set was then used for further verification. It turned out that the survival time in the high-risk group was significantly lower than that in the low-risk group (Figure 3(a)). ROC curves presented that the AUC values of 1-year, 3-year, and 5-year survivals were all about 0.7, indicting the accurate model performance (Figure 3(b)). To sum up, a risk model composed of 10 lncRNAs was constructed to evaluate the prognosis of ccRCC patients. The validation results showed that the model was accurate and showed good diagnostic efficiency.

### 3.3. The 10-lncRNA-Based Risk Model Is Independent in Predicting Prognosis

**Figure 4:**
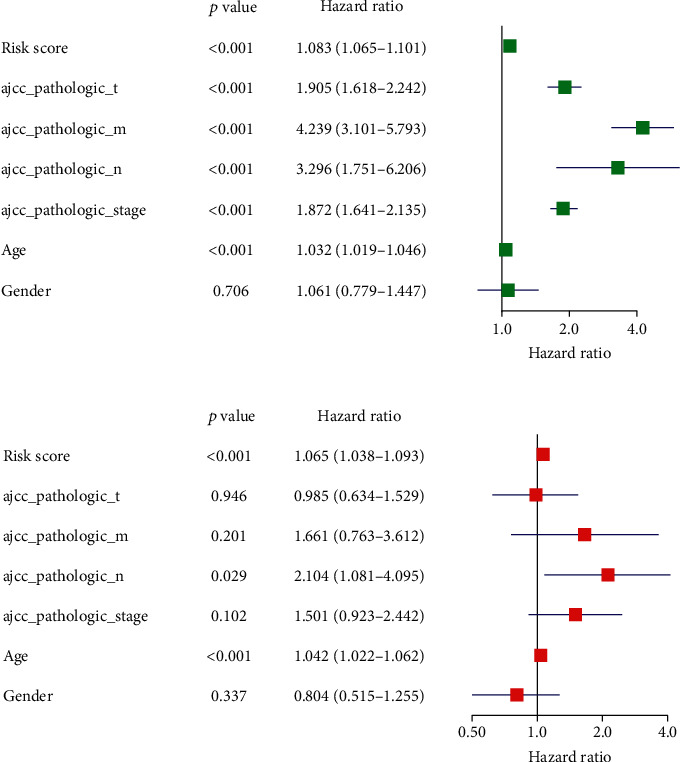
Univariate and multivariate analysis of prognostic factors. (a) Univariate analysis of prognostic factors; (b) multivariate analysis of prognostic factors.

In order to verify whether the 10-lncRNA risk model is independent in predicting prognosis, the model-based risk score plus clinical characteristics was analyzed in Cox regression analysis. Univariate regression analysis showed that age, pathologic_T, pathologic_N, pathologic_M, clinical stage, and the risk score were significantly correlated with the prognosis of patients (Figure 4(a)). Multivariate regression analysis showed that only age and the risk score were significantly correlated to the prognosis of patients (Figure 4(b)). The results showed that the risk score based on the 10-lncRNA signature was capable of independently predicting the prognosis of ccRCC patients.

### 3.4. Nomogram Establishment and Verification

**Figure 5:**
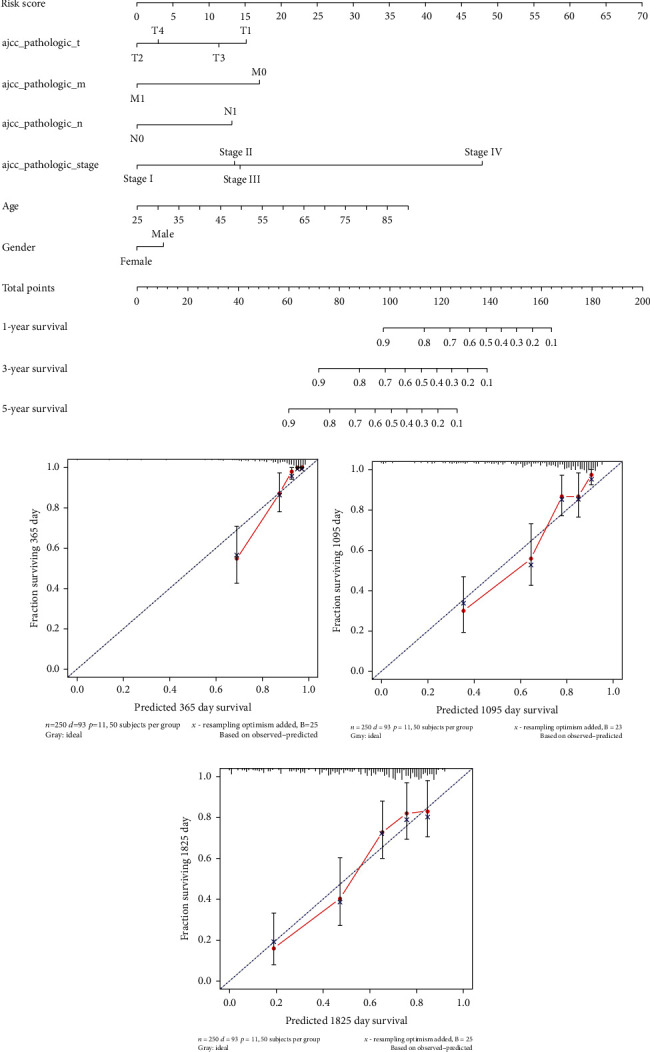
Nomogram for clinical prediction of survival time. (a) Nomogram for prediction of 1-year, 3-year, and 5-year survival times; (b–d) fitting curves of the nomogram for 1-year, 3-year, and 5-year survival times.

Since the risk model enabled independent prediction of the prognosis of patients, a nomogram for verification was drawn. The nomogram combining clinical indicators and the1 0-lncRNA-based risk score could be used to assist clinical diagnosis (Figure 5(a)). After establishment, the accuracy of the nomogram was assessed by fitting curves, and the results showed that the nomogram showed good fitness (Figures 5(b)–5(d)). Based on the above results, it is believed that the nomogram was accurate in predicting the survival time of ccRCC patients.

### 3.5. GSEA Enrichment Analysis in the High- and Low-Risk Groups

**Figure 6:**
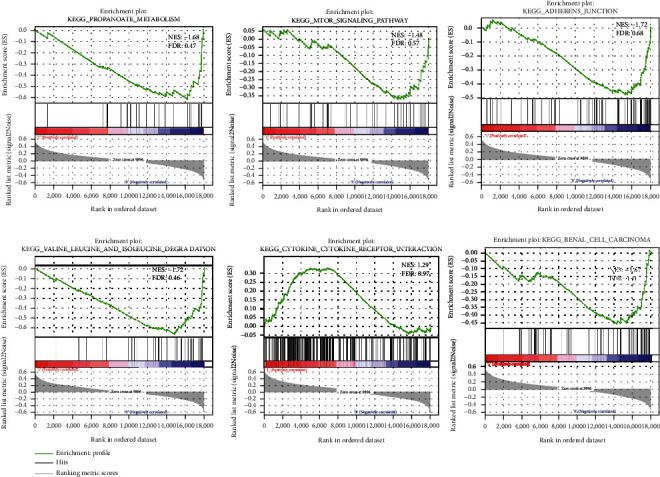
GSEA enrichment analysis for the high- and low-risk groups.

In order to explore the reason for the difference in prognosis between the high-risk group and the low-risk group, GSEA software was used to analyze the pathway enrichment between the two groups. The results showed that pathways involved in propanoate metabolism, mTOR signaling pathway, cell adhesion, cytokine receptor interaction, and renal cell carcinoma were significantly different (Figure 6). It turned out that in the high-risk group, the metabolism of tumor tissue was active, the immunity was inhibited, and the EMT was activated. Based on the above results, it is believed that the poor prognosis of ccRCC patients in the high-risk group may be related to the changes in activity of the above pathways.

## 4. Discussion

ccRCC is one of the most common types of cancer. In the United States, ccRCC causes nearly 64,000 new cancer cases and more than 13,000 deaths per year [36]. Biomarkers based on gene expression can help to improve the accuracy of early diagnosis and prognosis prediction. In recent years, many biomarkers are verified to predict the prognosis of patients, and many of them have the potential to predict the clinical prognosis of ccRCC patients. For example, MGAT5 is a potential independent prognostic biomarker in ccRCC patients after nephrectomy [37]. The promoter methylation of PCDH8 is associated with poor prognosis in ccRCC [38]. Yao and other experts evaluated the biological functions of CADM1-AS1 with miRNA and found that CADM1-AS1 is a new tumor suppressor in ccRCC [39]. Moreover, this lncRNA is correlated with poor prognosis of ccRCC. Xue and other researchers used qRT-PCR assay to detect the expression of NBAT-1 in ccRCC cell lines and analyzed the correlation between NBAT-1 and clinicopathological features. This study found that NBAT-1 expression in ccRCC tissue and RCC cells is significantly lower than that in normal tissue and normal cells, and this low level is associated with poor prognosis [40]. Although a large number of biomarkers with clinical significance were detected by experiments, most studies focus on a single biomarker or small number of samples, and the results lack the support of clinical data. Based on TCGA database, this study analyzed the data of lncRNAs related to ccRCC for screening a ccRCC prognostic lncRNA signature.

Many studies screened lncRNAs related to the prognosis of ccRCC, including LOC389332, SPRY4-IT1, and MFI2-AS114, and constructed prognostic models [41–44]. In this study, ccRCC patients were divided into five groups according to *K*-means clustering, and then, the differences in gene expression of each group were analyzed. Univariate Cox regression, LASSO regression, and multivariate regression analyses were used to identify new lncRNA markers for ccRCC prognosis. Finally, 10 lncRNAs (KIF9_AS1, GSEC, LIN00894, TNFRSF14_AS1, AC147651.4, AGAP2_AS1, RNF144A_AS1, AC008556.1, AL137127.1, and HLA_DQB1_AS1) were obtained. As analyzed, the patients with lowly expressed TNFRSF14_AS1 and AL137127.1 had a better prognosis and the ones with highly expressed KIF9_AS1, GSEC, LIN00894, AC147651.4, AGAP2_AS1, RNF144A_AS1, AC008556.1, and HLA_DQB1_had a worse prognosis. The role of these genes in ccRCC has not been reported except KIF9_AS1 and AGAP2_AS1. However, most of them are closely related to the development of multiple cancers. For example, TNFRSF14_AS1 is considered to be associated with breast cancer occurrence [45]. AC147651.4 is considered a biomarker of lung cancer [46]. RNF144A_AS1 is shown to enhance the migration of lymphoma [47]. In addition, KIF9_AS1 is believed to induce drug resistance of ccRCC patients by regulating TGF-*β* [48]. Gao and other experts found that the prognosis of ccRCC patients with high expression of AGAP2_AS1 is poor [49]. In addition to the above lncRNAs, the other five lncRNAs have not been reported, and their role in cancer needs to be further verified in the future. In conclusion, these prognosis-related lncRNAs with biological functions in cancer may be important targets for a further study of ccRCC.

After screening prognosis-related genes, we constructed a lncRNA-based prognostic model and verified its effectiveness in prognosis prediction. The results showed that the risk model could accurately predict the prognosis of patients in an independent manner. Thereafter, we also combined the risk model with clinical features for comprehensive analysis and built a nomograph to assist prediction. Finally, the functions of the 10 lncRNAs were explored. Enrichment analysis showed that the prognosis of patients was related to the changes of the pathways involved in metabolism, immunity, mTOR, and cell adhesion. It is reported that activity of metabolism-related pathways is closely related to tumor growth. A study found that the activity of amino acid metabolism-related pathways can promote the progression of ccRCC [50]. Cytokines and chemokines are important pathways affecting antitumor immunoreaction, and ccRCC may achieve immune escape by affecting the activity of related pathways. In addition, mTOR is considered to be an important pathway to promote tumor growth, and dysregulated expression of mTOR can promote tumor proliferation and metabolism [51]. In conclusion, it is believed that the changes in pathways related to metabolism, immunity and mTOR are the reason for the difference in prognosis of ccRCC patients.

This study is aimed at identifying lncRNAs which may be related to the prognosis of ccRCC with bioinformatics methods. A ccRCC prognostic risk model consisting of 10 lncRNAs was established, and the ability to predict prognosis was evaluated. The results showed that these 10 lncRNAs could be used as biomarkers for ccRCC diagnosis and can provide references for cancer diagnosis and prognosis. In addition, since the above results are only based on bioinformatics mining of TCGA database, more experimental data were needed for validation.

## Data Availability

The data used to support the findings of this study are included within the article. The data and materials in the current study are available from the corresponding author on reasonable request.

## Supplementary Materials

Supplementary Materials Supplementary Table 1: patient's clinical data.
