# Supplementary material for: Effect of Aberrant Long Noncoding RNA on the Prognosis of Clear Cell Renal Cell Carcinoma
Source: Comput Math Methods Med. 2021 Sep 3;2021:6533049. doi: 10.1155/2021/6533049 (PMC8433025; doi:10.1155/2021/6533049)
Supplement: Supplementary Materials — Supplementary Table 1: patient's clinical data. [file 6533049.f1.pdf]

submitter_	days_to_la	case_id	gender	race	vital_statu	age_at_inc	days_to_d	years_smo
TCGA-BP-	1384	cdd8eb5c	male	white	Alive	47	NA	NA
TCGA-CJ-	2231	9f4cd3b9-	male	white	Alive	61	NA	NA
TCGA-B0-	1169	c5bf474c-	female	white	Alive	72	NA	NA
TCGA-BP-	1011	ffb88267-	male	white	Alive	61	NA	NA
TCGA-CZ-	2439	a43401ae-	female	white	Alive	59	NA	NA
TCGA-AK-	1143	52e9daf0-	male	black or a	Alive	48	NA	NA
TCGA-A3-	1478	36ed3269	male	white	Alive	51	NA	NA
TCGA-CJ-	NA	4edff57f-4	female	white	Dead	54	224	NA
TCGA-B0-	NA	5ec1ec7b-	female	white	Dead	68	510	NA
TCGA-B8-	519	540cbfa7-	female	black or a	Alive	49	NA	NA
TCGA-B0-	NA	123b1a31	male	white	Dead	72	106	NA
TCGA-B0-	NA	6a7a0f51-	female	white	Dead	66	238	NA
TCGA-BP-	406	f217a4f9-	male	white	Alive	54	NA	NA
TCGA-GK-	61	0df9ec46-	female	black or a	Alive	76	NA	NA
TCGA-B0-	NA	949736aa	male	white	Dead	55	139	NA
TCGA-CZ-	NA	2efcefca-5	male	white	Dead	68	1404	NA
TCGA-BP-	NA	a5d34ea3	male	white	Dead	74	334	NA
TCGA-A3-	NA	0c139772	male	not report	Dead	57	1191	NA
TCGA-CZ-	2789	c4247ccb-	male	white	Alive	43	NA	NA
TCGA-B0-	2461	8f8a632d-	male	white	Alive	65	NA	NA
TCGA-BP-	205	19cd6d5b	male	white	Alive	76	NA	NA
TCGA-CJ-	3302	ec3b2a30	female	white	Alive	60	NA	NA
TCGA-B2-	507	fc7065fc-	male	black or a	Alive	61	NA	46
TCGA-EU-	206	68a1a23a	male	white	Alive	55	NA	NA
TCGA-BP-	411	b15f495d-	male	white	Alive	52	NA	NA
TCGA-B0-	18	8942ebfa-	male	white	Dead	68	18	NA
TCGA-AK-	3409	fdda296a-	female	white	Alive	48	NA	NA
TCGA-CZ-	2422	467bf226-	male	white	Alive	57	NA	NA
TCGA-BP-	NA	2dde6b93	male	white	Dead	42	1343	NA
TCGA-T7-	356	df1e2e79-	female	black or a	Alive	47	NA	NA
TCGA-CJ-	1855	4467ae73	male	white	Dead	77	3615	NA
TCGA-B0-	NA	e9f855b3-	male	white	Dead	33	222	NA
TCGA-CZ-	NA	305eaef4-	female	white	Dead	70	166	NA
TCGA-B4-	16	1bc2ad71	female	white	Alive	64	NA	NA
TCGA-B2-	992	08a8eec3-	male	white	Alive	59	NA	NA
TCGA-CW	3222	4100b960	male	white	Alive	60	NA	NA
TCGA-CJ-	1416	b7719c71	male	white	Alive	48	NA	NA
TCGA-CJ-	1759	4fd166d4-	female	white	Alive	72	NA	NA
TCGA-B0-	NA	ac30d54e	male	white	Dead	49	2090	NA
TCGA-CJ-	1657	a8db1341	male	white	Alive	61	NA	NA
TCGA-CZ-	1789	e9faa588-	male	white	Alive	69	NA	NA
TCGA-BP-	1126	3cfe743c-	male	white	Alive	53	NA	NA
TCGA-BP-	1955	ff9328d5-	male	white	Alive	64	NA	NA
TCGA-B0-	NA	11111b58	male	white	Dead	71	480	NA
TCGA-BP-	1746	1e417b93	male	white	Alive	40	NA	NA
TCGA-CZ-	NA	d5213422	male	white	Dead	56	1432	NA
TCGA-B2-	755	597c81a3-	male	white	Alive	74	NA	NA
TCGA-CJ-	NA	33cd8b05	female	white	Dead	69	1714	NA
TCGA-B0-	NA	df3b1215-	female	white	Dead	72	77	NA
TCGA-A3-	1186	4fca10bd-	male	white	Alive	51	NA	NA
TCGA-B0-	NA	0bd313f0-	male	white	Dead	54	883	NA
TCGA-A3-	1559	c7e9ba09	female	white	Alive	60	NA	NA
TCGA-CJ-	NA	3fa6c93e-	male	white	Dead	73	679	NA
TCGA-B8-	53	ea456a1f-	male	black or a	Alive	50	NA	NA
TCGA-BP-	932	5c73b91a	male	asian	Alive	49	NA	NA
TCGA-B2-	963	7d35b1e4	male	white	Alive	56	NA	NA
TCGA-B4-	11	aa82fb26-	male	white	Alive	45	NA	NA

TCGA-CJ-	3451	5338d435	male	white	Alive	64	NA	NA
TCGA-A3-	1130	22b6724c-	male	white	Alive	70	NA	NA
TCGA-BP-	501	a5790b81	male	white	Alive	66	NA	NA
TCGA-CJ-	3987	960d1eaf-	male	white	Alive	51	NA	NA
TCGA-BP-	1433	d97ac1d1	male	white	Alive	40	NA	NA
TCGA-AK-	3328	b3a6acab	female	white	Alive	54	NA	NA
TCGA-CJ-	3341	c1b46fd8-	female	white	Alive	79	NA	NA
TCGA-A3-	1385	0bbfeec1-	female	white	Alive	34	NA	NA
TCGA-B8-	150	7e620e32-	male	black or a	Alive	48	NA	NA
TCGA-B8-	511	cba5705a-	female	white	Alive	41	NA	NA
TCGA-B0-	NA	1b0ad45e	female	white	Dead	73	1724	NA
TCGA-CZ-	3271	b10eb61b	male	white	Alive	46	NA	NA
TCGA-BP-	970	a8e0f80a-	female	white	Alive	51	NA	NA
TCGA-B0-	NA	4ee14e95-	male	white	Dead	60	313	NA
TCGA-CJ-	1946	f964511a-	female	white	Alive	63	NA	NA
TCGA-B0-	NA	18bcef21-	female	white	Dead	46	578	NA
TCGA-B0-	1782	171044a9-	male	white	Alive	54	NA	NA
TCGA-B0-	NA	77d8c2cf-	male	white	Dead	63	204	NA
TCGA-BP-	NA	31201e73-	male	white	Dead	62	162	NA
TCGA-CZ-	NA	f2801b21-	male	white	Dead	63	722	NA
TCGA-B8-	NA	dfa9513d-	female	white	Dead	66	709	NA
TCGA-BP-	1632	2375ef64-	male	white	Alive	77	NA	NA
TCGA-A3-	1886	c04c5ac6-	male	white	Alive	41	NA	NA
TCGA-AK-	2531	fd5728e0-	female	white	Alive	58	NA	NA
TCGA-BP-	NA	f9e6afeb-	male	white	Dead	61	375	NA
TCGA-B0-	3631	d26f3a6b-	male	black or a	Alive	77	NA	NA
TCGA-CJ-	1450	e45166cb-	male	white	Alive	47	NA	NA
TCGA-B8-	505	7aece0e0-	female	black or a	Alive	38	NA	NA
TCGA-B8-	469	a2663a86-	male	black or a	Alive	61	NA	NA
TCGA-CZ-	662	8e9e684c-	male	white	Alive	76	NA	NA
TCGA-MM	700	aa2b6825-	female	black or a	Alive	58	NA	NA
TCGA-B0-	NA	3be0bc1d	female	black or a	Dead	53	1584	NA
TCGA-A3-	1307	8a575e00-	female	white	Alive	57	NA	NA
TCGA-A3-	1508	50f29fc7-	female	white	Alive	52	NA	NA
TCGA-BP-	693	2f38a984-	female	white	Alive	50	NA	NA
TCGA-MM	607	620baefa-	male	black or a	Alive	68	NA	NA
TCGA-BP-	749	d4098753	male	white	Alive	75	NA	NA
TCGA-A3-	1621	2bc5b2ec-	female	white	Alive	54	NA	NA
TCGA-B0-	NA	31e9d2fc-	male	white	Dead	63	1378	NA
TCGA-BP-	1413	62613d1d	female	asian	Alive	67	NA	NA
TCGA-BP-	211	33cf0893-	male	white	Alive	42	NA	NA
TCGA-BP-	620	38469030-	female	white	Alive	74	NA	NA
TCGA-B0-	4074	db058eb4	female	white	Alive	47	NA	NA
TCGA-B0-	2722	514af471-	female	black or a	Alive	68	NA	NA
TCGA-B0-	NA	0022478c-	male	white	Dead	53	563	NA
TCGA-A3-	861	46c87ae0-	male	white	Alive	52	NA	NA
TCGA-CJ-	NA	73c712fe-	female	white	Dead	64	139	NA
TCGA-BP-	951	54c9850c-	male	white	Alive	63	NA	NA
TCGA-A3-	2270	69fb3eff-a	male	white	Alive	72	NA	NA
TCGA-B2-	648	cfc999aa-	male	white	Alive	52	NA	NA
TCGA-G6-	NA	3f0a2571-	female	black or a	Dead	54	242	NA
TCGA-BP-	967	b9fc9eff-1	male	white	Alive	79	NA	NA
TCGA-BP-	1893	453085d8	male	white	Alive	47	NA	NA
TCGA-BP-	2372	02b5012e	male	white	Alive	50	NA	NA
TCGA-B8-	16	07aa333c-	female	black or a	Alive	65	NA	NA
TCGA-AK-	1168	d7cf4fc0-	male	white	Alive	48	NA	NA
TCGA-BP-	2718	e7bbc8a9-	male	white	Alive	59	NA	NA
TCGA-EU-	119	4481ca64-	female	white	Alive	67	NA	NA

TCGA-B0-	NA	616bc43c-	male	white	Dead	81	245	NA
TCGA-BP-	1291	64aa0dd1	male	white	Alive	57	NA	NA
TCGA-CZ-	1929	9cdda9fa-	male	white	Alive	74	NA	NA
TCGA-BP-	2080	80c4fc5b-	male	white	Alive	78	NA	NA
TCGA-B0-	1552	fedcaa7b-	female	white	Alive	61	NA	NA
TCGA-DV	1329	f6d28a1d-	male	white	Alive	59	NA	NA
TCGA-BP-	1489	9816ac2a-	male	white	Alive	48	NA	NA
TCGA-B0-	NA	7a4283eb	male	white	Dead	46	101	NA
TCGA-DV	2470	01277e9d	male	black or a	Alive	55	NA	NA
TCGA-BP-	1876	dc6f2273-	male	white	Alive	63	NA	NA
TCGA-A3-	1436	7ac1d6c6-	male	not report	Alive	66	NA	NA
TCGA-BP-	NA	39867756-	male	white	Dead	52	2454	NA
TCGA-BP-	NA	9f5c0a43-	male	white	Dead	63	878	NA
TCGA-CJ-	3498	852effcd-	female	white	Alive	60	NA	NA
TCGA-B0-	NA	e7c8a4d3-	male	white	Dead	64	1598	NA
TCGA-A3-	2688	4d856b5d	male	white	Alive	62	NA	NA
TCGA-BP-	2964	b81243b3	female	white	Alive	64	NA	NA
TCGA-CJ-	750	d65f7f50-	female	white	Alive	76	NA	NA
TCGA-BP-	NA	3f05a719-	female	white	Dead	76	701	NA
TCGA-A3-	910	ee7011e7-	male	white	Alive	42	NA	NA
TCGA-B0-	NA	cf67eaf8-	female	white	Dead	72	68	NA
TCGA-BP-	NA	57959b73	male	white	Dead	57	1493	NA
TCGA-BP-	2184	6dc3788e-	male	white	Alive	43	NA	NA
TCGA-CZ-	2067	ea26d89f-	male	white	Alive	51	NA	NA
TCGA-B0-	2150	fb9bafa5-	female	white	Alive	61	NA	NA
TCGA-B0-	NA	44130745-	male	white	Dead	49	1371	NA
TCGA-BP-	NA	c499b3be	male	white	Dead	75	62	NA
TCGA-B4-	38	afd60992-	male	white	Alive	59	NA	NA
TCGA-B0-	NA	fb3a387f-	female	white	Dead	90	0	NA
TCGA-B0-	NA	03b2ac94-	male	white	Dead	77	1317	NA
TCGA-CJ-	2014	24c1cf70-	male	white	Alive	41	NA	NA
TCGA-B0-	NA	1380d4cc-	male	white	Dead	88	454	NA
TCGA-B0-	2172	74844571-	male	white	Alive	71	NA	NA
TCGA-B0-	1290	d7ab7ec0	male	white	Alive	64	NA	NA
TCGA-CJ-	1528	900b5f21-	male	asian	Alive	42	NA	NA
TCGA-BP-	2881	844d8b94	male	white	Alive	67	NA	NA
TCGA-BP-	785	ccd44d97-	male	white	Alive	75	NA	NA
TCGA-B8-	256	3280a078-	female	black or a	Alive	69	NA	NA
TCGA-AK-	1217	d7abeadd	male	white	Alive	83	NA	NA
TCGA-CJ-	NA	267ff78b-	male	white	Dead	64	93	NA
TCGA-B0-	NA	bcbab8ce-	male	white	Dead	74	110	NA
TCGA-A3-	689	52db2d93	female	black or a	Alive	49	NA	NA
TCGA-B0-	NA	9cffe9f2-4	female	white	Dead	74	2764	NA
TCGA-BP-	1133	e9847c37-	male	white	Alive	36	NA	NA
TCGA-A3-	2274	9f632fc3-	female	white	Alive	48	NA	NA
TCGA-A3-	751	9868ae73-	male	white	Dead	52	1170	NA
TCGA-CJ-	1793	f11f68ac-	female	white	Alive	67	NA	NA
TCGA-B0-	3974	bf768635-	female	white	Alive	62	NA	NA
TCGA-B0-	NA	d599ff9b-	male	white	Dead	51	182	NA
TCGA-B8-	1525	a1ad1a96-	male	black or a	Alive	42	NA	NA
TCGA-B8-	1476	59278f79-	male	white	Alive	71	NA	NA
TCGA-CW	2799	2d0f6d4f-	male	white	Alive	44	NA	NA
TCGA-BP-	1888	fdfaf862-	female	black or a	Alive	60	NA	NA
TCGA-BP-	1935	d23d354d	male	white	Alive	47	NA	NA
TCGA-DV	370	16559faa-	male	white	Alive	26	NA	NA
TCGA-BP-	293	8f54d854-	female	white	Alive	46	NA	NA
TCGA-BP-	2172	0d88e6ab	male	white	Alive	46	NA	NA
TCGA-A3-	1137	2e81cfda-	male	white	Alive	47	NA	NA

TCGA-CJ-	646	2309c424	male	white	Dead	42	646	NA
TCGA-BP-	1124	aebe37c4	female	asian	Alive	41	NA	NA
TCGA-B0-	3834	326745d8	male	white	Alive	53	NA	NA
TCGA-B4-	166	70820391	male	white	Alive	52	NA	NA
TCGA-B0-	NA	57578a0d	male	white	Dead	48	1417	NA
TCGA-BP-	NA	ce0ab696	female	white	Dead	75	1097	NA
TCGA-A3-	1993	4c44f4a4-	female	white	Alive	46	NA	NA
TCGA-BP-	NA	6e9e5e05	male	white	Dead	71	1912	NA
TCGA-CZ-	NA	6949efc1-	male	white	Dead	60	206	NA
TCGA-CJ-	NA	5db69ebe	female	white	Dead	65	768	NA
TCGA-B0-	NA	420409bb	male	white	Dead	58	168	NA
TCGA-A3-	735	11ab53c8	male	white	Alive	64	NA	NA
TCGA-CJ-	NA	eee108d6	male	white	Dead	84	1972	NA
TCGA-CJ-	NA	cdfa3c5d-	female	white	Dead	55	1661	NA
TCGA-MN	591	fe1f95a3-	male	black or a	Alive	41	NA	NA
TCGA-B0-	NA	04004bc7	male	white	Dead	57	320	NA
TCGA-G6-	474	6b033bf5-	female	black or a	Dead	62	1091	NA
TCGA-A3-	1257	a55e63a9	female	white	Alive	86	NA	NA
TCGA-B0-	4537	62cf9546-	female	white	Alive	65	NA	NA
TCGA-CZ-	685	5722df9f-	male	not report	Alive	67	NA	NA
TCGA-BP-	1462	7b184674	female	white	Alive	43	NA	NA
TCGA-B8-	1218	e7afc830-	male	white	Alive	56	NA	NA
TCGA-BP-	NA	ba96288c	female	white	Dead	51	992	NA
TCGA-CJ-	3519	46d34d0c	male	white	Alive	72	NA	NA
TCGA-BP-	1854	63dd7202	female	white	Alive	67	NA	NA
TCGA-B0-	NA	18524601	female	white	Dead	77	885	NA
TCGA-A3-	1493	12c4c4f1-	male	white	Alive	57	NA	NA
TCGA-CW	2378	58ef1a13-	male	white	Alive	52	NA	NA
TCGA-B0-	3944	9c377f9b-	female	white	Alive	66	NA	NA
TCGA-CJ-	NA	c4bbbcd2	male	white	Dead	48	932	NA
TCGA-BP-	1516	8958c2bf-	male	white	Alive	62	NA	NA
TCGA-AK-	369	0a93c52e	female	black or a	Alive	71	NA	NA
TCGA-CW	2609	0487fc41-	male	white	Alive	51	NA	NA
TCGA-B0-	NA	414056a2	female	white	Dead	80	1639	NA
TCGA-CJ-	1520	9fe336a8-	male	white	Alive	61	NA	NA
TCGA-A3-	617	9dc7812b	male	white	Alive	49	NA	NA
TCGA-BP-	NA	f8804530-	male	white	Dead	70	2601	NA
TCGA-BP-	1879	830626c5	male	white	Alive	49	NA	NA
TCGA-A3-	1314	0b970feb-	female	black or a	Alive	51	NA	NA
TCGA-AK-	2865	3c5f27e9-	male	white	Alive	58	NA	NA
TCGA-CJ-	NA	8fd493c5-	female	white	Dead	48	336	NA
TCGA-BP-	1502	2a9c4ce5-	female	white	Alive	43	NA	NA
TCGA-CJ-	1498	78048432	female	black or a	Alive	58	NA	NA
TCGA-DV	365	fef57c51-(	male	black or a	Alive	53	NA	NA
TCGA-B8-	528	09d7ce7a	male	black or a	Alive	60	NA	NA
TCGA-B0-	1604	46bc69a9	male	white	Alive	43	NA	NA
TCGA-BP-	NA	9b84d013	female	white	Dead	75	109	NA
TCGA-BP-	NA	e04bd954	female	white	Dead	79	1270	NA
TCGA-B4-	7	ab5266f6-	female	white	Alive	61	NA	NA
TCGA-A3-	0	c33d0d12	female	black or a	Alive	65	NA	NA
TCGA-BP-	1413	89500416	male	white	Alive	54	NA	NA
TCGA-B0-	2246	576ea0ef-	male	white	Alive	73	NA	NA
TCGA-CZ-	330	393abcf7-	male	white	Dead	52	330	NA
TCGA-CW	2552	4dd51edf-	male	white	Alive	68	NA	NA
TCGA-CJ-	2038	0631dc8c	female	white	Alive	59	NA	NA
TCGA-B4-	175	551e8a06	male	white	Alive	62	NA	NA
TCGA-B8-	1046	e58f2c7b-	female	white	Alive	41	NA	NA
TCGA-AK-	1853	351fb9f4-	female	white	Dead	62	2241	NA

TCGA-DV	355	fc052a9d-	female	white	Alive	29	NA	NA	
TCGA-BP-	NA	34e8fbd8-	female	white	Dead	70	562	NA	
TCGA-BP-	1459	b81998f2-	male	white	Alive	59	NA	NA	
TCGA-CZ-	3267	291f45ab-	female	white	Alive	79	NA	NA	
TCGA-CZ-	1905	13e25128-	male	white	Alive	60	NA	NA	
TCGA-B0-	NA	12adefc4-	male	white	Dead	58	866	NA	
TCGA-BP-	182	270c9a0b-	male	white	Alive	57	NA	NA	
TCGA-CJ-	2186	7ca06dbe-	female	white	Alive	71	NA	NA	
TCGA-BP-	NA	c3cc716b-	female	white	Dead	73	329	NA	
TCGA-BP-	1063	f1ed9155-	male	white	Alive	44	NA	NA	
TCGA-B0-	NA	f57690ba-	male	white	Dead	63	342	NA	
TCGA-A3-	NA	2304b8f8-	male	white	Dead	74	561	NA	
TCGA-CW	2271	f1ae0181-	male	white	Alive	56	NA	NA	
TCGA-B8-	1299	b428463a	female	white	Alive	51	NA	NA	
TCGA-CJ-	3229	3e976301-	female	white	Alive	49	NA	NA	
TCGA-B8-	909	59d18f0c-	female	black or a	Alive	62	NA	NA	
TCGA-CZ-	59	878d1caa-	male	white	Dead	84	59	NA	
TCGA-B0-	NA	cf77fe39-(	male	white	Dead	70	1986	NA	
TCGA-B0-	1778	75801b19	male	white	Alive	57	NA	NA	
TCGA-A3-	323	a42103aa-	male	black or a	Alive	37	NA	NA	
TCGA-B8-	762	a22d5ba2	male	white	Alive	74	NA	NA	
TCGA-EU-	551	2de427e2	female	white	Alive	47	NA	NA	
TCGA-DV	1130	c6454371-	male	white	Alive	41	NA	NA	
TCGA-B4-	141	59f49821-	female	white	Alive	61	NA	NA	
TCGA-BP-	354	9a404208-	female	white	Alive	55	NA	NA	
TCGA-BP-	932	265f1d03-	male	white	Alive	60	NA	NA	
TCGA-B0-	2782	c91a4cc3-	female	white	Alive	75	NA	NA	
TCGA-B2-	952	bbbce1ba	male	white	Alive	61	NA	NA	
TCGA-BP-	645	e5eca191-	male	white	Dead	56	645	NA	
TCGA-BP-	193	2d7e2ae4-	male	white	Alive	70	NA	NA	
TCGA-BP-	29	b6d4e2d5	male	white	Alive	75	NA	NA	
TCGA-B0-	NA	46194f7f--	female	white	Dead	77	637	NA	
TCGA-BP-	1843	c22d25c2-	female	white	Alive	55	NA	NA	
TCGA-B2-	972	1adb05d4	male	white	Alive	83	NA	NA	
TCGA-B0-	NA	98ff8a6b-	male	white	Dead	81	99	NA	
TCGA-BP-	1367	a9f73fb6-	male	white	Alive	74	NA	NA	
TCGA-CW	2017	7b4e529f-	female	white	Alive	78	NA	NA	
TCGA-B0-	3989	88c91a7b-	male	white	Alive	50	NA	NA	
TCGA-B0-	1411	f6d3e963-	male	white	Alive	46	NA	NA	
TCGA-BP-	NA	57070e5b	male	white	Dead	52	1092	NA	
TCGA-B8-	523	78ec8bc9-	male	white	Alive	58	NA	NA	
TCGA-AK-	2392	c0171d4e-	male	white	Alive	69	NA		20
TCGA-CZ-	NA	f5759059-	male	white	Dead	60	445	NA	
TCGA-BP-	1355	d30f460b-	male	white	Alive	58	NA	NA	
TCGA-BP-	NA	79e469c5-	male	white	Dead	64	1912	NA	
TCGA-BP-	1107	dd35edfd-	male	white	Alive	34	NA	NA	
TCGA-BP-	2412	30208a4c-	male	white	Alive	55	NA	NA	
TCGA-BP-	563	2a2f168c-	male	white	Alive	40	NA	NA	
TCGA-BP-	372	f72defb3-	female	white	Alive	68	NA	NA	
TCGA-CJ-	1952	c0f7147e-	female	white	Alive	42	NA	NA	
TCGA-BP-	1140	e548aeb2	male	white	Alive	45	NA	NA	
TCGA-A3-	1610	3372a7d2	female	white	Dead	76	1610	NA	
TCGA-CJ-	NA	e865d40a	male	white	Dead	90	0	NA	
TCGA-BP-	1177	d6ea5cec-	female	white	Alive	43	NA	NA	
TCGA-B0-	NA	844ec449-	male	white	Dead	60	793	NA	
TCGA-CJ-	NA	06197e26-	female	white	Dead	46	431	NA	
TCGA-CJ-	1531	07f596f7-	male	white	Alive	38	NA	NA	
TCGA-CJ-	2283	804a6159-	female	white	Alive	73	NA	NA	

TCGA-B2-	656	a682a2a3-	male	white	Alive	79	NA	NA
TCGA-BP-	1133	3128d9bf-	male	white	Alive	54	NA	NA
TCGA-AS-	43	8593764c-	male	white	Alive	35	NA	NA
TCGA-AS-	1238	e3aa5595-	male	white	Alive	63	NA	NA
TCGA-B0-	2009	f3a921d5-	female	white	Alive	71	NA	NA
TCGA-CZ-	1997	4c474c70-	male	white	Alive	58	NA	NA
TCGA-B0-	2430	bb4a6123	male	white	Alive	57	NA	NA
TCGA-CJ-	NA	2890b330	male	white	Dead	58	1625	NA
TCGA-B0-	NA	8d4b602b	female	white	Dead	76	202	NA
TCGA-B0-	NA	6b1ff32f-7	female	white	Dead	65	927	NA
TCGA-BP-	2208	44143697-	male	white	Alive	40	NA	NA
TCGA-CZ-	25	577847b9	male	white	Dead	67	2419	NA
TCGA-A3-	630	798d51ff-	male	white	Alive	60	NA	NA
TCGA-A3-	574	e1663694-	male	white	Alive	69	NA	NA
TCGA-BP-	1371	9245a557-	male	white	Alive	68	NA	NA
TCGA-CJ-	NA	f2fca062-(	male	white	Dead	59	1567	NA
TCGA-BP-	1124	a68bd706	male	white	Alive	57	NA	NA
TCGA-CW	2226	73fc6ae6-	female	white	Alive	62	NA	NA
TCGA-B0-	NA	5b66c4ae-	male	white	Dead	75	42	NA
TCGA-B8-	777	eda2b871	female	white	Alive	70	NA	NA
TCGA-A3-	1018	2953131e-	male	black or af	Alive	47	NA	NA
TCGA-BP-	1625	7664c241-	female	white	Dead	53	1625	NA
TCGA-B0-	NA	4cfe9a35-	male	white	Dead	63	600	NA
TCGA-AK-	874	28e9b6f9-	male	white	Alive	84	NA	NA
TCGA-B8-	435	828ad452	female	white	Alive	67	NA	NA
TCGA-B8-	1520	18325fdc-	female	white	Alive	63	NA	NA
TCGA-BP-	1834	c129b63f-	male	white	Alive	63	NA	NA
TCGA-CJ-	3736	4c325ee1-	male	white	Alive	60	NA	NA
TCGA-A3-	1624	21eea125-	male	white	Alive	75	NA	NA
TCGA-B4-	155	75e02a42-	male	white	Alive	65	NA	NA
TCGA-AK-	1423	d7155ce6-	male	white	Alive	45	NA	NA
TCGA-A3-	1491	8ee720b5	male	not report	Alive	67	NA	NA
TCGA-B0-	NA	ff127be4-	male	white	Dead	65	1588	NA
TCGA-BP-	1308	82f069c4-	male	white	Alive	54	NA	NA
TCGA-DV	2004	aba26e6b	female	black or af	Alive	40	NA	NA
TCGA-CJ-	841	d1e4f4c0-	male	white	Dead	58	841	NA
TCGA-B0-	NA	58dee518	male	white	Dead	61	1238	NA
TCGA-CZ-	1787	dc43cfe0-	female	white	Alive	59	NA	NA
TCGA-CJ-	1906	f2b93fbd-	male	white	Alive	54	NA	NA
TCGA-CJ-	1776	53dede5a	female	white	Alive	85	NA	NA
TCGA-CJ-	3205	942c0088-	male	white	Alive	47	NA	NA
TCGA-CW	2489	7080bc0a-	female	white	Alive	51	NA	NA
TCGA-AK-	3583	97fee782-	male	white	Alive	65	NA	NA
TCGA-CW	NA	bbdaa931	male	white	Dead	61	41	NA
TCGA-CZ-	1446	5e248b21	female	not report	Dead	76	2564	NA
TCGA-EU-	127	28c8c2c6-	male	white	Alive	81	NA	NA
TCGA-BP-	1794	3b064b7e	female	white	Alive	63	NA	NA
TCGA-BP-	13	3c4ed50e-	male	white	Alive	69	NA	NA
TCGA-B0-	NA	efb088d9-	female	white	Dead	69	834	NA
TCGA-BP-	840	b5f51cf6-1	male	white	Alive	61	NA	NA
TCGA-B0-	NA	d99f31f7-	male	white	Dead	52	1200	NA
TCGA-MV	498	b123e7c1-	female	black or af	Alive	72	NA	NA
TCGA-BP-	1670	c15d5920-	male	white	Alive	65	NA	NA
TCGA-BP-	2746	d9f1bb02-	male	white	Alive	74	NA	NA
TCGA-BP-	1018	4c3e4727-	male	white	Alive	53	NA	NA
TCGA-DV	1729	0ff579a1-(	female	white	Alive	52	NA	NA
TCGA-CZ-	NA	b602a73b	female	white	Dead	86	73	NA
TCGA-BP-	1785	b3a9b0be	male	white	Alive	58	NA	NA

TCGA-A3-	735	10c03d66-	male	black or a	Dead	59	735	NA
TCGA-CJ-	NA	d2664fde-	female	white	Dead	54	782	NA
TCGA-A3-	945	d4815225	male	white	Alive	79	NA	NA
TCGA-BP-	NA	83bdd67b	female	white	Dead	72	2343	NA
TCGA-B2-	1092	424e5c99-	male	white	Alive	73	NA	NA
TCGA-CZ-	1315	7fc6b44d-	male	white	Dead	86	1315	NA
TCGA-CZ-	1928	88e7ce26-	female	white	Alive	51	NA	NA
TCGA-CZ-	NA	226ce515-	male	white	Dead	63	446	NA
TCGA-CJ-	1496	830b8de5	female	white	Alive	62	NA	NA
TCGA-CZ-	2754	f00b7956-	male	white	Alive	62	NA	NA
TCGA-CJ-	3480	61c75ffb-	male	white	Alive	49	NA	NA
TCGA-B0-	26	26cc2c84-	male	white	Dead	61	65	NA
TCGA-B0-	3744	0a6a375b	female	white	Alive	39	NA	NA
TCGA-BP-	NA	c0e26587-	male	white	Dead	79	2256	NA
TCGA-BP-	NA	23c7555b	male	white	Dead	60	822	NA
TCGA-A3-	468	96c38b74-	female	black or a	Alive	57	NA	NA
TCGA-BP-	204	382291d3	male	white	Alive	79	NA	NA
TCGA-BP-	NA	d6625c4d	male	white	Dead	58	211	NA
TCGA-CZ-	373	883c37e2-	male	white	Alive	61	NA	NA
TCGA-BP-	NA	d9208e7b	male	white	Dead	40	1034	NA
TCGA-DV	NA	936c217f-	female	black or a	Dead	55	727	NA
TCGA-BP-	603	8f936d84-	male	white	Alive	72	NA	NA
TCGA-CJ-	4067	310c31bf-	male	white	Alive	47	NA	NA
TCGA-B8-	495	f2b90375-	female	black or a	Alive	54	NA	NA
TCGA-A3-	1385	8d02978a	male	white	Alive	79	NA	NA
TCGA-3Z-	385	2b1dea0a	male	black or a	Alive	69	NA	NA
TCGA-B0-	1733	62f5ceb7-	female	white	Alive	59	NA	NA
TCGA-DV	2016	ea854858-	male	white	Alive	37	NA	NA
TCGA-BP-	1014	fae200ed-	male	white	Alive	42	NA	NA
TCGA-B0-	1790	4e3334e2-	male	white	Alive	77	NA	NA
TCGA-CJ-	1435	820ddb1a	male	white	Alive	51	NA	NA
TCGA-B0-	183	52d80054	female	white	Dead	60	183	NA
TCGA-6D-	362	d3b47e53	female	black or a	Alive	68	NA	NA
TCGA-B8-	822	d4159990	female	white	Alive	63	NA	NA
TCGA-BP-	NA	ffed886e-	male	white	Dead	76	1111	NA
TCGA-CJ-	1521	83444c79-	female	white	Alive	65	NA	NA
TCGA-B0-	1485	73512f9d-	male	white	Alive	56	NA	NA
TCGA-A3-	1106	e4da964a	male	white	Alive	53	NA	NA
TCGA-B0-	3205	3b2b492b	male	white	Alive	45	NA	NA
TCGA-BP-	177	6ccd4657-	male	white	Alive	58	NA	NA
TCGA-A3-	1120	9fb55e0b-	male	white	Alive	67	NA	NA
TCGA-B8-	830	9256867c-	male	black or a	Alive	69	NA	NA
TCGA-BP-	374	9ecdde9c-	female	asian	Alive	62	NA	NA
TCGA-CJ-	1955	e002c248-	male	white	Alive	57	NA	NA
TCGA-CJ-	NA	d232f22d-	female	white	Dead	57	819	NA
TCGA-BP-	NA	e719b7f3-	male	white	Dead	70	1133	NA
TCGA-B0-	NA	74fc9f65-	male	white	Dead	63	1045	NA
TCGA-BP-	1071	b472c982-	male	white	Alive	46	NA	NA
TCGA-B2-	919	ed2e9354	male	white	Alive	79	NA	NA
TCGA-CZ-	NA	74749fe8-	male	white	Dead	63	561	NA
TCGA-CW	NA	88fc4bc4-	female	white	Dead	73	1964	NA
TCGA-AK-	3728	9da334ed	male	white	Alive	62	NA	NA
TCGA-B0-	3392	794aeb92	female	white	Alive	53	NA	NA
TCGA-CZ-	NA	e0127e51-	male	white	Dead	83	311	NA
TCGA-B8-	788	65d56c51-	male	black or a	Alive	63	NA	NA
TCGA-B0-	NA	8d769b38	female	asian	Dead	68	1230	NA
TCGA-B8-	737	89ea18fc-	male	white	Alive	43	NA	NA
TCGA-B0-	NA	bcf26561-	female	white	Dead	88	485	NA

TCGA-B0-	3841	c814c26c-	male	white	Alive	53	NA	NA	
TCGA-BP-	2263	c558dffe-!	male	white	Alive	53	NA	NA	
TCGA-AK·	2217	08c1bb22	male	white	Alive	72	NA	NA	
TCGA-A3·	1070	46240f32-	male	black or a	Dead	51	1696	NA	
TCGA-CJ-	2423	dbe16232	male	white	Alive	63	NA	NA	
TCGA-B0-	2630	dfd2c288-	male	white	Alive	50	NA	NA	
TCGA-BP-	2361	4cfe4f31-1	male	black or a	Alive	69	NA	NA	
TCGA-BP-	1165	315b6dea	male	white	Alive	56	NA	NA	
TCGA-CJ-	NA	772e3aed·	female	white	Dead	52	2227	NA	
TCGA-AK·	3343	1b3f2411-	male	white	Alive	68	NA	NA	
TCGA-BP-	1862	db601d82	female	white	Alive	54	NA	NA	
TCGA-B0-	NA	f184de71-	female	white	Dead	75	2145	NA	
TCGA-B0-	3431	31a0dd95	female	white	Alive	66	NA	NA	
TCGA-BP-	118	dc2208d9	male	white	Alive	58	NA	NA	
TCGA-B0-	NA	8aaa4e25·	male	white	Dead	62	333	NA	
TCGA-CJ-	NA	822cf6c1-	male	black or a	Dead	62	574	NA	
TCGA-B0-	NA	3f72d63f-	male	white	Dead	76	1337	NA	
TCGA-B0-	1608	ae55b2d3	male	white	Alive	40	NA	NA	
TCGA-CZ·	NA	6205c2d8·	male	white	Dead	41	946	NA	
TCGA-CJ-	1560	692f9978-	male	white	Alive	50	NA	NA	
TCGA-B0-	NA	9ceefc7e-	male	black or a	Dead	72	1913	NA	
TCGA-CW	NA	5f53ace3-	male	white	Dead	32	571	NA	
TCGA-BP-	2660	5003a0cb·	male	white	Alive	49	NA	NA	
TCGA-B0-	NA	e33dff22-	male	white	Dead	60	1980	NA	
TCGA-B0-	NA	3dd36758	male	white	Dead	47	478	NA	
TCGA-B8-	26	766999d7	male	white	Alive	65	NA	NA	
TCGA-DV	NA	ef2d7ef5-·	male	black or a	Dead	59	1626	NA	
TCGA-A3·	340	2711b4a4	male	black or a	Alive	75	NA		65
TCGA-B0-	NA	62c6edc7·	female	white	Dead	76	770	NA	
TCGA-BP-	1132	d3638a1f-	male	white	Alive	56	NA	NA	
TCGA-BP-	1666	513a4de3	female	white	Alive	75	NA	NA	
TCGA-B0-	665	03ae8fdb-	female	white	Alive	59	NA	NA	
TCGA-BP-	NA	c285ada3·	male	white	Dead	72	952	NA	
TCGA-B0-	NA	a46f36c3-	male	white	Dead	78	1111	NA	
TCGA-A3·	567	8ed08632	male	not report	Alive	54	NA	NA	
TCGA-CJ-	1924	2d8da564	male	white	Alive	51	NA	NA	
TCGA-B0-	NA	9d202594	male	white	Dead	51	69	NA	
TCGA-B0-	NA	b764e6a1	male	white	Dead	81	1019	NA	
TCGA-G6·	2133	1d37becb	female	black or a	Alive	81	NA		40
TCGA-BP-	1266	9aa63985·	male	white	Alive	56	NA	NA	
TCGA-B0-	NA	479035f0-	female	white	Dead	79	362	NA	
TCGA-CZ·	2128	6b79d20c	male	white	Alive	69	NA	NA	
TCGA-DV	1398	4b2ec8aa·	female	white	Alive	67	NA	NA	
TCGA-CW	3146	3f93d1cf-a	male	white	Alive	73	NA	NA	
TCGA-B8-	722	ab954d5e	female	white	Alive	61	NA	NA	
TCGA-AK·	1508	0709749f-	female	white	Alive	85	NA	NA	
TCGA-CZ·	386	99f59583-	female	not report	Alive	72	NA	NA	
TCGA-CJ-	3936	ceb7cd9a·	male	white	Alive	70	NA	NA	
TCGA-A3·	3	060b2104	male	black or a	Alive	78	NA	NA	
TCGA-BP-	NA	781acafa-	male	white	Dead	75	1463	NA	
TCGA-AK·	NA	2ed3296a	female	white	Dead	71	683	NA	
TCGA-B0-	NA	1e758394·	male	white	Dead	79	307	NA	
TCGA-CJ-	NA	2939c03a·	female	white	Dead	44	552	NA	
TCGA-B8-	1525	b125ff14-·	male	white	Alive	57	NA	NA	
TCGA-CZ·	774	86f68b7d-	male	white	Alive	82	NA	NA	
TCGA-BP-	NA	8be7d2e2	male	white	Dead	67	1589	NA	
TCGA-BP-	400	8d5d69a4	female	white	Alive	72	NA	NA	
TCGA-B0-	2609	1d176c53·	male	white	Alive	69	NA	NA	

TCGA-A3-	NA	897f85ec-	male	white	Dead	68	137	NA
TCGA-CJ-	1889	740e8238-	male	white	Alive	78	NA	NA
TCGA-B0-	1755	4298ccdb-	female	white	Alive	75	NA	NA
TCGA-CW	NA	9fd9104b-	male	white	Dead	51	1075	NA
TCGA-CJ-	2554	06d86d68	male	white	Alive	49	NA	NA
TCGA-CZ-	18	fcecafa8-8	female	white	Alive	62	NA	NA
TCGA-B8-	194	98dea82b	male	white	Alive	53	NA	NA
TCGA-BP-	714	5dc807f2-	male	white	Alive	59	NA	NA
TCGA-CJ-	1499	3972ae59-	male	white	Alive	58	NA	NA
TCGA-CZ-	2873	a7ddd737	male	white	Alive	55	NA	NA
TCGA-A3-	2504	e4cc8898-	female	white	Alive	82	NA	NA
TCGA-B0-	NA	03905b58	female	white	Dead	49	1657	NA
TCGA-BP-	NA	eeacbbdc-	female	white	Dead	59	953	NA
TCGA-BP-	NA	500636a5-	female	white	Dead	59	480	NA
TCGA-B8-	1380	8c6720ef-	female	white	Alive	73	NA	NA
TCGA-BP-	1487	e173c4ba-	male	white	Alive	40	NA	NA
TCGA-CW	NA	09c4ea05-	male	white	Dead	74	164	NA
TCGA-B0-	NA	d51fa004-	female	white	Dead	53	459	NA
TCGA-B0-	1274	b6abbdcc	male	white	Alive	52	NA	NA
TCGA-BP-	2839	e7c159b7-	female	white	Alive	60	NA	NA
TCGA-A3-	3	b39d6675	female	black or a	Alive	59	NA	NA
TCGA-CJ-	2353	818022ea-	male	white	Dead	67	3554	NA
TCGA-B0-	NA	759238f2-	female	white	Dead	78	1121	NA
TCGA-AK-	2508	5cf9d9fe-	male	white	Alive	58	NA	NA
TCGA-BP-	NA	ed97aeee	female	white	Dead	65	475	NA
TCGA-CZ-	693	f2806652-	male	white	Alive	38	NA	NA
TCGA-B0-	NA	e4769374-	female	white	Dead	82	2386	NA
TCGA-BP-	2257	028d32fc-	female	white	Alive	45	NA	NA
TCGA-A3-	873	136c95cd-	male	white	Alive	46	NA	NA
TCGA-BP-	3074	0242658f-	female	white	Alive	65	NA	NA
TCGA-BP-	454	cc353686-	male	white	Alive	57	NA	NA
TCGA-BP-	3037	3c1b6de7-	female	white	Alive	64	NA	NA
TCGA-B2-	NA	52d73201	female	black or a	Dead	72	51	NA
TCGA-BP-	845	6ccabcd0-	male	white	Dead	75	845	NA
TCGA-CJ-	1883	3ea341e3-	male	black or a	Alive	57	NA	NA
TCGA-A3-	0	f38a6799-	female	black or a	Alive	74	NA	NA
TCGA-BP-	2859	4978eb8d	male	white	Alive	43	NA	NA
TCGA-B0-	NA	b7143068	male	white	Dead	69	587	NA
TCGA-CJ-	NA	6ab00314	male	white	Dead	65	2299	NA
TCGA-AK-	2087	17d053f2-	male	white	Alive	72	NA	NA
TCGA-CZ-	1943	f06bdea2-	male	white	Dead	39	2105	NA
TCGA-B8-	431	04beccb3-	female	black or a	Alive	60	NA	NA
TCGA-BP-	1871	ec6aa756-	male	white	Alive	46	NA	NA
TCGA-CJ-	NA	b7994839	male	white	Dead	62	1200	NA
TCGA-BP-	1885	a50fe35a-	female	white	Alive	57	NA	NA
TCGA-AK-	NA	c5265ca6-	male	white	Dead	37	885	NA
TCGA-G6-	305	28011111-	male	black or a	Dead	55	313	NA
TCGA-A3-	319	659294b9	male	asian	Alive	50	NA	NA
TCGA-CJ-	1373	4519a839-	female	white	Alive	69	NA	NA
TCGA-B4-	365	d0f908c5-	female	white	Alive	68	NA	NA
TCGA-B8-	36	d62d39f9-	male	white	Alive	62	NA	NA
TCGA-B0-	1175	439794a8-	female	white	Alive	69	NA	NA
TCGA-A3-	16	3cbca837-	female	white	Alive	77	NA	NA
TCGA-BP-	1731	6fbf13b2-	male	white	Alive	46	NA	NA
TCGA-AK-	3331	b2feca3f-8	male	white	Alive	40	NA	NA
TCGA-BP-	NA	2913faf2-	female	white	Dead	76	2	NA
TCGA-BP-	408	5daf4808-	male	white	Alive	39	NA	NA
TCGA-CZ-	1683	6b0c1e54-	male	white	Alive	63	NA	NA

TCGA-B2-	417	73cb1350·	male	white	Dead	46	1003	NA	
TCGA-B0-	NA	5f0f03c2-‹	male	white	Dead	65	43	NA	
TCGA-BP-	433	c01afa58-	male	asian	Alive	44	NA	NA	
TCGA-CJ-	3639	beef6a78-	female	white	Alive	63	NA	NA	
TCGA-BP-	NA	037c691e·	female	white	Dead	74	344	NA	
TCGA-BP-	1495	5358b8c7·	female	white	Alive	58	NA	NA	
TCGA-AK·	2868	fa543627-	male	white	Alive	48	NA		15
TCGA-BP-	NA	9490cd1e·	female	white	Dead	78	1590	NA	
TCGA-BP-	3377	1cdb170b	male	white	Alive	69	NA	NA	

ked
